# Integration of Radiomics and Tumor Biomarkers in Interpretable Machine Learning Models

**DOI:** 10.3390/cancers15092459

**Published:** 2023-04-25

**Authors:** Lennart Brocki, Neo Christopher Chung

**Affiliations:** Institute of Informatics, University of Warsaw, Banacha 2, 02-097 Warsaw, Poland

**Keywords:** deep learning, artificial intelligence, interpretability, explainability, concept bottleneck, radiomics, tumor biomarkers, feature engineering, feature selection

## Abstract

**Simple Summary:**

Artificial intelligence (AI) based on deep neural networks (DNNs) has demonstrated great performance in computer vision. However, their clinical application in the diagnosis and prognosis of cancer using medical imaging has been limited. Not knowing the AI’s decision-making process (interpretability) presents a major obstacle in AI medical applications. To this end, we studied and propose the integration of DNN-derived biomarkers and expert-derived radiomics in interpretable ConRad models. ConRad models achieved great performance for malignancy classification while maintaining inherent interpretability. Without interpretability, a black box classifier such as end-to-end DNNs may harbor critical failure modes that are unknown and unknowable.

**Abstract:**

Despite the unprecedented performance of deep neural networks (DNNs) in computer vision, their clinical application in the diagnosis and prognosis of cancer using medical imaging has been limited. One of the critical challenges for integrating diagnostic DNNs into radiological and oncological applications is their lack of interpretability, preventing clinicians from understanding the model predictions. Therefore, we studied and propose the integration of expert-derived radiomics and DNN-predicted biomarkers in interpretable classifiers, which we refer to as ConRad, for computerized tomography (CT) scans of lung cancer. Importantly, the tumor biomarkers can be predicted from a concept bottleneck model (CBM) such that once trained, our ConRad models do not require labor-intensive and time-consuming biomarkers. In our evaluation and practical application, the only input to ConRad is a segmented CT scan. The proposed model was compared to convolutional neural networks (CNNs) which act as a black box classifier. We further investigated and evaluated all combinations of radiomics, predicted biomarkers and CNN features in five different classifiers. We found the ConRad models using nonlinear SVM and the logistic regression with the Lasso outperformed the others in five-fold cross-validation, with the interpretability of ConRad being its primary advantage. The Lasso is used for feature selection, which substantially reduces the number of nonzero weights while increasing the accuracy. Overall, the proposed ConRad model combines CBM-derived biomarkers and radiomics features in an interpretable ML model which demonstrates excellent performance for lung nodule malignancy classification.

## 1. Introduction

Cancer kills about 10 million people annually and is a leading cause of death worldwide. Lung cancer is the most prevalent type of cancer in the world [1] and kills more patients than any other cancer in the United States [2]. Individuals suspected of or suffering from cancer routinely undergo medical imaging acquisition using computed tomography (CT), positron emission tomography (PET), and other modalities, whose data (e.g., pixels and voxels) are becoming increasingly larger and more complex. Despite deep neural networks showing unprecedented performance in computer vision, their application to medical imaging in clinical routine has been limited [3,4]. One of the most important issues is a lack of interpretable machine learning models and a domain-specific implementation whose prediction can be understood by and communicated to clinicians [5].

**Figure 1:**
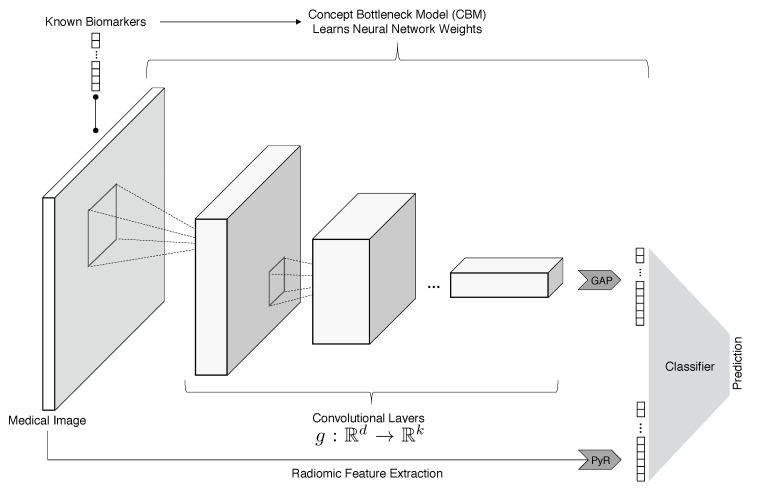
The proposed ConRad model integrates expert-derived radiomics features [6,7] and biomarkers predicted from a concept bottleneck model (CBM) [10]. The final interpretable classifier uses both types of features competitively to classify a tumor as benign or malignant.

To overcome this challenge in the context of lung nodule malignancy classification, we have developed and investigated an interpretable machine learning model called ConRad that combines concept bottleneck models (CBMs), which predict tumor biomarkers, and radiomics features, which are based on expert-derived characterization of medical images (Figure 1). Both of these feature sets have the advantage that their meaning is clearly defined which enhances our model’s transparency. Radiomics [6,7] has been used to identify cancer signatures that are visually indistinguishable for doctors and to discern cancer subtypes [8]. On the other hand, deep neural networks (DNNs) may result in high-performance classifiers or features that cannot be understood or are not explainable by oncological experts. As a proof of concept, we applied and evaluated different aspects of ConRad using the LIDC-IDRI (Lung Image Database Consortium and Image Database Resource Initiative) dataset [9].

Importantly, instead of using the annotated biomarkers directly in the malignancy prediction, we followed the concept bottleneck model (CBM) architecture [10] and trained a DNN to predict the biomarkers which were then used in the final classifier. Therefore, once our model has been trained, biomarker annotations, which are expensive and time-consuming to obtain, are no longer needed. Additionally, we performed standard radiomics feature extraction which provides interpretable statistical properties of cancer tumors. Both feature sets are fused to train the final classifier to predict benign versus malignant tumors. Feature selection was explored by applying the Lasso (least absolute shrinkage and selection operator) [11] to the logistic regression. Lasso has been shown to maintain, or even increase, the model performance while utilizing a substantially small number of features.

We have performed a comprehensive evaluation of various machine learning (ML) algorithms in ConRad models. Through five-fold cross-validation, the performances were measured in terms of model accuracy, receiver operating characteristic (ROC) curves, and other metrics. For a baseline comparison, we also built an end-to-end convolutional neural network (CNN), which directly predicts malignancy. From the end-to-end CNN, the CNN features are extracted and used in independent ML classifiers for further comparison. Both the end-to-end CNN and the usage of CNN features represent uninterpretable black-box models since radiologists do not gain any understanding of how the model is making its prediction. Our interpretable models perform comparably or better than the CNN. There is an inherent trade-off between interpretability and accuracy in machine learning, including DNNs [12]. Instead of hyper-optimizing DNN architectures on LIDC-IDRI data, our study aimed to engineer inherently interpretable features that are based on well-established radiological and oncological expertise.

In the next Section 2, we summarize the related works, focusing on the development of radiomics and DNNs for oncology. Our ConRad model, data, and evaluation are detailed in Section 3. Section 4 shows the performance metrics of different ML classifiers and the comparison against black-box classifiers based on CNNs. We also demonstrate informative feature selection, automatically achieved through the use of the Lasso. Finally, we summarize our findings and provide concluding remarks in Section 5.

## 2. Related Works

While the comprehensive review of DNNs applied on the lung nodule classification is beyond the scope of this paper, we note a few related analyses of the LIDC-IDRI dataset [9]; see Table 1 for the most relevant ones. Two of the earliest examples are [13,14], which use a supervised multiscale approach and unsupervised feature extraction via an autoencoder, respectively. Other approaches include 3D CNNs, which have been used by [15], so-called dilated CNN introduced in [16], and curriculum learning [17], where the model is first trained on easy and later on harder samples, progressively growing the network in the process. What all these approaches have in common is that they focus on improving model accuracy and do not consider the interpretability of the developed models.

Our model shares similarities with the methods employed in [18,19,20], which all use features beyond the image pixels themselves. Radiomics features are combined with CNN features in [18], but in contrast to our method, the annotated biomarkers are not used. Biomarkers are combined with CNN and radiomics features in the work of [19], but the biomarkers are not predicted by a computational model, and the annotations must be provided when the model is applied to unseen data. In [20], DNNs were used to predict biomarkers and to extract features from previous layers of the biomarker predictor to be used in the malignancy classifier via a jump connection. Those features entering the final malignancy classifier are not readily interpretable by humans.

Radiomics extracts high-dimensional expert-derived features from medical images, some of them being clinically validated and interpretable by clinicians [21]. There has been an international coordinated effort called the image biomarker standardization initiative (IBSI) to evaluate and standardize radiomics feature extraction [22], which we utilize in our pipeline. Radiologists and nuclear medicine doctors see radiomics as having the potential to provide a quantitative signature of tumors, that are often impossible to be detected by human experts [23]. Therefore, radiomics features have been used in the diagnosis and prognosis of multiple cancer types, including those of the breast [24,25], prostate [26], lung [27], head and neck [28], rectal [29], and others. Numerous studies have validated the predictive power of IBSI radiomics features for generalization to multiple cancer types.

## 3. Methods

### 3.1. ConRad Models and Data

The proposed ConRad model was designed to extract different aspects of cancer images, leveraging well-established statistical properties and clinical variables of tumors. Particularly, our final model uses biomarkers predicted from a concept bottleneck model (CBM) [10] and radiomics features [30] (visualized in Figure 1). CBM-predicted biomarkers (subtlety, calcification, sphericity, margin, lobulation, spiculation, texture, and diameter) and radiomics features are fed to ML classifiers to predict the tumor status (benign vs. malignant). Additionally, we built an end-to-end classifier based on a fine-tuned ResNet model [31] which we use as a baseline comparison.

We particularly focus on CT images of lung tumors from the LIDC-IDRI (Lung Image Database Consortium and Image Database Resource Initiative) dataset [9]. The dataset consists of thoracic CT scans of 1018 cases alongside segmentations, information on the likelihood of malignancy, and annotated biomarkers for nodules with diameter >3 mm obtained by up to four radiologists. The CT scans were processed using the pylidc package [30], which clusters the nodule annotations (This step is necessary in the presence of multiple nodules in a single scan since the dataset does not indicate which annotations belong to the same nodule. In case pylidc assigns more than four annotations to a nodule, the concerned nodule is not admissible) and provides a consensus consolidation of the annotated nodule contours. The likelihood of malignancy is calculated by taking the median of the radiologists’ annotations, which range from one (highly unlikely) to five (highly suspicious). Nodules with a median of three are discarded as ambiguous. Those with medians above or below three are labeled as benign or malignant, respectively. This procedure yielded a total of 854 nodules, with 442 being benign.

Input samples were created by first isotropically resampling the CT scans to 1 mm spacing and then extracting 32×32 crops around the nodule center in the axial, coronal, and sagittal planes. Hounsfield unit values below −1000 and above 400 were clamped to filter out air and bone regions.

### 3.2. Feature Engineering and Model Training

First, we conduct the radiomics feature extraction from LIDC-IDRI samples using the PyRadiomics package [21]. Particularly, three classes of radiomics features were calculated based on first-order statistics (18 features), 3D shapes (14 features), and higher-order statistics (75 features). All radiomics features were extracted from images where the tumor had been delineated using the segmentation masks provided by the LIDC data. First-order statistics including energy, entropy, centrality, and other distributional values were calculated. Shape features included volumes, areas, sphericity, compactness, elongation, and other descriptors of masked tumors. Higher-order statistics were given by the gray-level co-occurrence matrix, gray-level size zone, and others.

Second, a ResNet-50 [31] model pre-trained on the ImageNet [32] was fine-tuned to predict eight well-known clinical variables informative of tumors in a concept bottleneck model (CBM) [10]. Instead of predicting the tumor status directly (benign vs malignant), the CBM associates the input samples with annotated biomarkers provided by the LIDC-IDRI dataset; namely, subtlety, calcification, sphericity, margin, lobulation, spiculation, texture, and diameter. The values used for training the CBM are obtained by averaging the annotations provided by the different radiologists. To train the ResNet, we used PyTorch Lightning (https://www.pytorchlightning.ai/, accessed on 15 April 2023) and an Nvidia V100 GPU.

Note, that the degree to which the described features are interpretable, as well as in which way they are interpretable, varies. Radiomics features are all interpretable in the sense that they follow from an explicit and simple mathematical definition. Since many of them represent rather abstract statistical features, their meaning may nonetheless be not intuitive for clinicians. The predicted biomarkers, on the other hand, are computed by an opaque algorithm (the DNN), but their meaning can be immediately understood.

To match the input dimensions of the ResNet-50 model, input samples are upscaled to 224×224 and duplicated in each color channel. Input samples are z-normalized with the mean and standard deviation obtained from the training set, and identical normalization is applied to the test set. We employ 5-fold cross-validation. The model is trained for 50 epochs using the Adam optimizer with PyTorch default parameters and a minibatch size of 32. The initial learning rate is set to $10^{-3}$ and is annealed by multiplying by 0.1 after 20 and 40 epochs. During training, an input sample is obtained by randomly selecting one of the three views, and during testing the model output is averaged across all three views to obtain the final output. The trained CBM is used to obtain predicted values for selected biomarkers from samples in the testing set. Unless specified, our evaluation is based on predicted biomarkers (instead of annotated values) to reflect clinical practices where labor-intensive manual segmentation and quantification may be unavailable. Note that we included clinical labels from LIDC-IDRI, but depending on the data and domain experts, other biomarkers may be used to train the CBM.

Third, we also extracted features from training a convolutional neural network (CNN) to directly predict the tumor status. For a fair comparison, the same ResNet-50 architecture used in CBM was used to build the CNNs. The preprocessing and training procedure is identical to the CBM described above. This end-to-end classifier gives us a baseline to be compared with our proposed interpretable ConRad model. Furthermore, we extracted 512 features from the global average pooling layer of the fine-tuned ResNet-50, which are also fused with the radiomics features and CBM-derived biomarkers for downstream classification. We investigated whether a black box DNN model may have superior performance for classifying lung cancer images, and if so, what trade-off between interpretability and accuracy may exist and be acceptable for radiological applications.

### 3.3. Machine Learning Classifiers

There are three types of features available for training a model to classify the malignancy of nodules. Using each type of feature and their combinations, we constructed a total of 7 datasets for the final classifier:

- Biomarkers + radiomics (ConRad models);
- Radiomics features;
- Biomarkers (predicted by CBM);
- CNN features;
- CNN + radiomics;
- CNN + biomarkers;
- CNN + radiomics + biomarkers (all).

Note that the nodule diameter appears in both the biomarker and radiomics feature sets. Having two features that are closely related would cause instability. Thus, when necessary we removed the diameter from the biomarkers. All datasets were z-score normalized on the training set, and identical normalization was applied on the test set.

As final-layer classifiers (Figure 1), we applied and evaluated linear and nonlinear support vector machines (SVMs) [33], logistic regression [34] with and without Lasso regularization [11], and random forest [35]. We used the scikit-learn (https://scikit-learn.org, accessed on 15 April 2023) implementation of these classifiers. The SVM and Lasso regularization parameters *C* and λ, respectively, were selected via 5-fold cross-validation on the whole dataset. The Lasso (least absolute shrinkage and selection operator) adds a $L_{1}$ penalty to the loss function, which may achieve feature selection with coefficients of zero. The Lasso was applied to a large number of radiomics features to remove highly correlated features, such that the optimal model performance could be achieved with much fewer features [36]. For each combination of features, we track the number of features selected via the Lasso.

Overall, we built five classifiers using the seven aforementioned datasets, combing three types of features. We applied an independent 5-fold cross-validation, where each classifier was trained on four folds, and performance was evaluated on the hold-out fold. The performance metrics (namely accuracy, precision, and recall) were calculated and averaged across 5-fold cross-validation. A receiver operating characteristic (ROC) curve was constructed by measuring the false-positive rates (FPRs) and true-positive rates (TPRs) at a wide range of thresholds.

Our proposed model can be contrasted with popular end-to-end DNN algorithms which are considered to be uninterpretable “black box” models. We are interested in creating an interpretable method, where the final prediction is explainable by statistically and clinically relevant features. Furthermore, we leverage and expose oncological knowledge by directly utilizing radiomics features. Importantly, ML classifiers competitively use multiple types of features (radiomics and biomarkers) which helps to highlight their interplay.

## 4. Results

### 4.1. Evaluation of the ConRad Models

ConRad models combine tumor biomarkers predicted by DNNs and radiomics features defined by radiological experts. LIDC-IDRI contains 8 biomarkers, which were used to construct a concept bottleneck model (CBM) using the pre-trained ResNet-50. We ran each of the CT scans through PyRadiomics to obtain first-order statistics (18 features), 3D shapes (14 features), and higher- order statistics (75 features). We trained and evaluated five different ML classifiers based on a total of 115 features.

**Figure 2:**
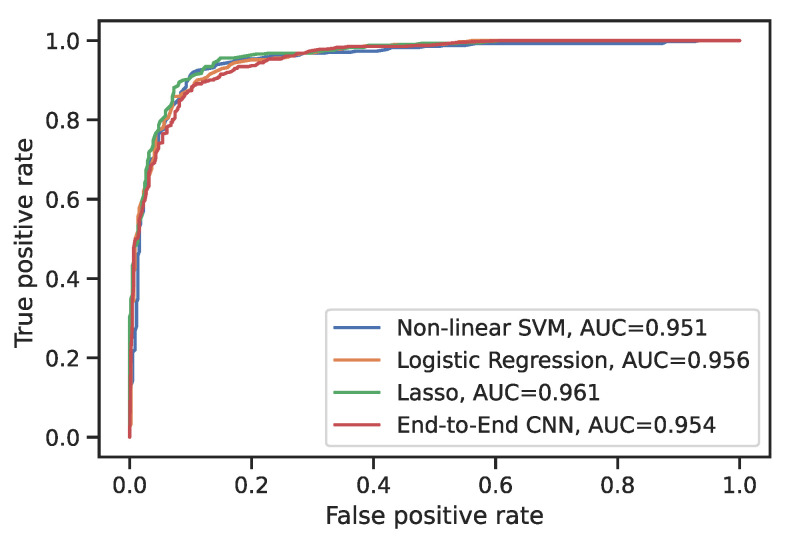
ROC curves for ConRad models in comparison with the end-to-end CNN model. At varying thresholds, false-positive and true-positive rates are measured for each classifier, followed by averaging over 5 test sets. The areas under the ROC curves (AUCs) are similar in all considered classifiers, with logistic regression with the Lasso slightly outperforming the others.

The model accuracy, recall, and precision were averaged over five-fold cross-validation, where the test set was not used in training. Table 2 shows that all the classifiers had an accuracy in the range of 0.881–0.897; the nonlinear (radial) SVM outperformed all other classifiers, whereas the random forest showed the worst performance. For the majority of feature sets, linear SVM performed worse than non-linear SVM. In terms of the ROC, all the ConRad models and the end-to-end CNN model performed similarly (Figure 2).

**Figure 3:**
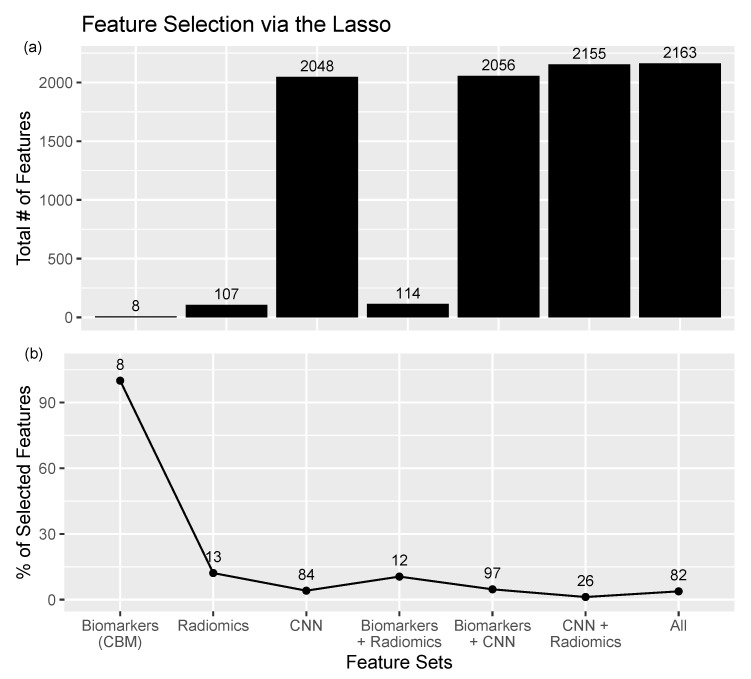
(**a**) Total number of features for each set of features. (**b**) Feature selection using the Lasso in logistic regressions. In each feature set, the Lasso ($L_{1}$ regularization) is applied with the penalty parameter selected via cross-validation. Except for biomarkers, which only contain 8 features, only small percentages of features are selected.

When the Lasso (least absolute shrinkage and selection operator) was additionally applied on logistic regression, only 12 out of 114 features (see “Biomarkers+Radiomics” in Figure 3) were selected. The logistic regression with the Lasso increased the model performance compared to that without the Lasso. Additionally, using a small set of features is inherently more interpretable, as 12 features can be readily visualized and understood.

### 4.2. Comparison to CNN Models

In order to compare the ConRad models to baselines, we constructed models that exclusively rely on CNN features, extracted from the end-to-end model outlined in Section 3. The end-to-end CNN classifier itself yielded an accuracy of 0.891, and the other evaluated classifiers had accuracies ranging from 0.858 to 0.891 (Table 3). CNN-based classifiers did, therefore, not perform as well as the ConRad models (see Table 2). Furthermore, radiomics and biomarkers are interpretable, representing morphological or biological characteristics, whereas CNN features are not interpretable. Note that ConRad does not aim to maximize the model accuracy, and even slightly lower accuracy may be acceptable in exchange for higher interpretability.

However, there is still a concern that uninterpretable CNN features may contain additional information that could increase the ConRad models’ performance. To test this idea, CNN features were added to ConRad models, creating the fullest models (“CNN + Radiomics + Biomarkers”). The five considered ML classifiers were trained on this feature set, and performances were measured over five-fold cross-validation (complete results are available in Appendix A Table A1). For all of the classifiers except random forest, adding CNN features on top of the radiomics and biomarkers decreased the accuracy. Table A1 shows that nonlinear SVM with the predicted biomarkers and radiomics (i.e., ConRad models) slightly outperformed the other approaches in terms of precision and accuracy. We also observed that the inclusion of the Lasso improved logistic regression for most feature sets across recall, precision, and accuracy measures.

### 4.3. The Lasso and Feature Selection

In a regression model, the Lasso [11] adds an $L_{1}$ penalty in the loss function which may reduce weights (i.e., coefficients in logistic regression), even to zero. The zero weights imply removal of their features, where only a remaining subset of features is used in classification. We found that the Lasso drastically reduced the number of considered features in most of the feature sets (Figure 3b). For all feature sets, the fraction of selected features was less than 15%, except for the biomarkers. All eight biomarkers were automatically selected by the Lasso, presumably due to their low dimension. In the consideration of the 2048 CNN features, only 4.10% (84 features) were selected (Figure 3).

Logistic regression with the Lasso did not only strongly reduce the number of selected features but also outperformed the nonregularized logistic regression on almost every considered dataset and performance metric (Table A1). On the pure radiomics features, the Lasso selects five shape features, seven higher-order statistics features, and the first-order minimum. With just 12.1% of the radiomics features, the accuracy remained the same. When combining biomarkers and radiomics features in the proposed ConRad approach, the Lasso selects five biomarkers and seven radiomics features. Using this subset of features (10.5% of features), the regularized logistic regression achieved a 0.896 accuracy, while the unregularized version had an 0.892 accuracy. Feature selection via the Lasso increases interpretability without sacrificing model performance.

## 5. Discussion

We investigated different models for classifying lung nodule malignancy, with a focus on interpretability. Particularly, as part of feature engineering, the concept bottleneck model (CBM) for predicting biomarkers was used. When applied, the proposed ConRad model does not need annotated biomarkers as they are usually not readily available in real world applications. In total, we investigated 35 classifiers, utilizing seven combinations of features and five ML algorithms. Additionally, an end-to-end CNN classifier was evaluated as a baseline. ConRad classifiers based on biomarkers and radiomics features showed excellent performance on par with the end-to-end CNN model, which inherently acts as a black box. Nonlinear SVM and logistic regression with the Lasso showed the best performance generally.

The intermediate features that feed into the final classifier are interpretable and clinically meaningful by design. The biomarkers represent properties of nodules that have an intuitive meaning for clinicians, who can therefore gain an understanding of the decision-making process of the model. This can nurture trust in the ML model since the users do not have to blindly believe the model’s classifications. Instead, the users can comprehend, and even interact with, how the model reaches its decisions. For example, if a truly small nodule has been incorrectly predicted to have a large diameter, the clinician may recognize this by visually inspecting the CT scan (i.e., routine task). This may allow the clinician to make informed decisions with the support of ML. Furthermore, in the ConRad model, the diameter prediction can be corrected by the clinician, directly resulting in an updated prediction. Our ConRad model, therefore, lends itself to a human-in-the-loop (HITL) approach, that may help gain a deeper understanding and trust of the model’s operating characteristics.

Besides using independent training steps to create an interpretable classifier, we also consider feature selection in malignancy classification. When combining biomarkers and radiomics features, the number of selected features was drastically reduced with the Lasso [11]. Many biomarkers and radiomics features may be correlated, and thus removing redundancy can stabilize the model and improve the overall performance with translational potentials [36]. When applied to CNN features, extreme reduction to only 1–5% of features actually improved the model accuracy. Not only are CNN features not interpretable, but a majority of them contain redundant information and do not contribute to the malignancy classification. In the future, this may be a fruitful way to investigate how to identify predictive features while ensuring the interpretability inherent in a manageable number of features.

The combination of biomarkers and radiomics features outperforms all other considered combinations, although the biomarkers demonstrated the most predictive power for the LIDC-IDRI data [9]. Some of the most critical information about lung nodules are contained in both feature sets, as exemplified by the diameter. Thus, we consider both feature sets to be a critical part of bringing clinical knowledge into interpretable ML, especially as real-world medical images may not contain as rich annotations as LIDC-IDRI. Predefined mathematical characterization of medical images in radiomics is always available and can be clinically validated [6,7]. We aim to investigate deeper into the interplay between these features, especially as we plan to apply ConRad to other medical imaging datasets.

## 6. Conclusions

Overall, the proposed ConRad model combines concept bottleneck models and radiomics to create an interpretable model. The model’s performance is on par with an end-to-end DNN classifier, despite the additional interpretability constraints. The increased transparency of our model can help radiologists and oncologists to understand its predictions, better allowing them to perform well-informed diagnoses. Without this critical interpretability component, a black box classifier such as an end-to-end DNN may harbor critical failure modes that are unknown and difficult to discover. Therefore, instead of focusing solely on model performance for medical applications, more investigations that consider interpretability are warranted to realize the broader incorporation of explainable AI into radiology and oncology.

## Figures and Tables

**Table 1:** Overview of previous deep learning approaches to the LIDC-IDRI dataset.

Reference	Summary
[13]	Crops at multiple scales are fed to CNNs with shared parameters, and extracted features are concatenated for final classification
[18]	Radiomics features are combined with CNN features
[19]	Biomarkers, radiomics, and CNN features combined, and no model is trained to predict biomarkers
[20]	Biomarkers are predicted with a CNN, but intermediate features with no well-defined meaning are used in the final prediction

**Table 2:** Evaluation of different classifiers in the ConRad models. Performance metrics are averaged over the five-fold cross-validation.

Final Layer Classifier	Recall	Precision	Accuracy
Non-linear SVM	0.886	0.899	0.897
Linear SVM	0.886	0.893	0.893
Random Forest	0.879	0.883	0.881
Logistic Regression	0.884	0.893	0.892
Logistic Regression with the Lasso	0.896	0.893	0.896

**Table 3:** Model accuracy of the baseline CNN models. The first row indicates an end-to-end CNN classifier. Other classifiers use CNN features, in comparison to Table 2.

Classifier	Accuracy
End-to-end CNN	0.891
Nonlinear SVM	0.875
Linear SVM	0.87
Random Forest	0.888
Logistic Regression	0.858
Logistic Regression with the Lasso	0.891

**Figure A1:**
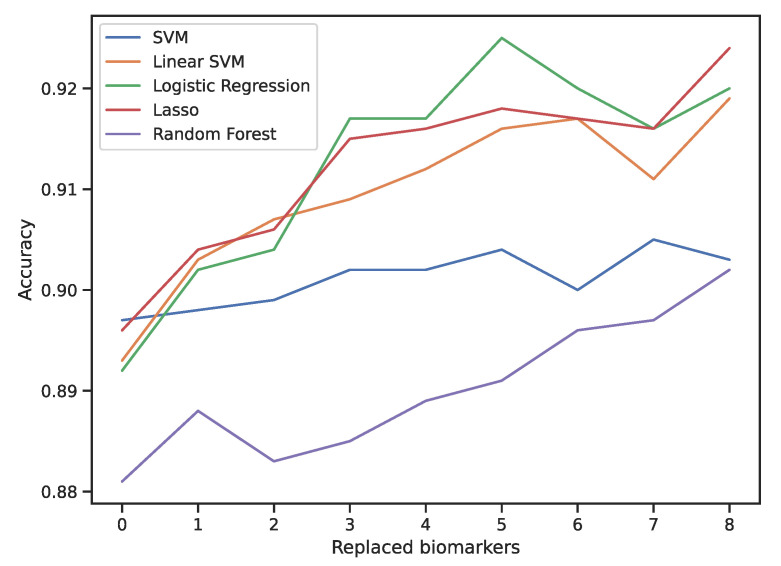
Change in accuracy when the models were retrained after replacement of the predicted biomarkers with annotated ones. Biomarkers were replaced in order from the least to most correctly predicted ones.

## Data Availability

The code to reproduce the results are available at https://github.com/lenbrocki/ConRad, accessed on 12 April 2023. The lung cancer data from LIDC-IDRI dataset are available at https://wiki.cancerimagingarchive.net/pages/viewpage.action?pageId=1966254, accessed on 12 April 2023.

## Abbreviations

The following abbreviations are used in this manuscript:

DNN	deep neural network
CNN	convolutional neural network
CBM	concept bottleneck model
LIDC-IDRI	Lung Image Database Consortium and Image Database Resource Initiative
CT	computerized tomography
PET	positron emission tomography
Lasso	least absolute shrinkage and selection operator
SVM	support vector machine
ROC	receiver operating characteristic
AUC	area under the curve
FPR	false-positive rate
TPR	true-positive rate

## Appendix A

**Table A1:** Full comparison of five classifiers on 7 different combinations of features. Performance metrics are averaged over the five-fold cross-validation.

Classifier	Features	Recall	Precision	Accuracy
Nonlinear SVM	CNN	0.869	0.880	0.875
	Radiomics	0.869	0.873	0.876
	Biomarkers	0.900	0.876	0.890
	All	0.869	0.886	0.879
	CNN+rad	0.869	0.878	0.876
	Bio+rad	0.886	0.899	0.897
	Bio+CNN	0.876	0.868	0.891
Linear SVM	CNN	0.859	0.862	0.870
	Radiomics	0.879	0.880	0.883
	Biomarkers	0.891	0.893	0.896
	All	0.855	0.870	0.863
	CNN+rad	0.864	0.873	0.868
	Bio+rad	0.886	0.893	0.893
	Bio+cnn	0.852	0.861	0.869
Random forest	CNN	0.871	0.887	0.888
	Radiomics	0.876	0.878	0.878
	Biomarkers	0.891	0.879	0.889
	All	0.881	0.897	0.885
	CNN+rad	0.874	0.894	0.890
	Bio+rad	0.879	0.883	0.881
	Bio+cnn	0.891	0.889	0.890
Logistic regression	CNN	0.857	0.858	0.858
	Radiomics	0.874	0.884	0.883
	Biomarkers	0.886	0.892	0.893
	All	0.871	0.878	0.875
	CNN+rad	0.864	0.864	0.874
	BIO+rad	0.884	0.893	0.892
	BIO+cnn	0.850	0.867	0.874
Logistic regression with the Lasso (feature selection)	CNN	0.871	0.876	0.891
	Radiomics	0.876	0.881	0.883
	Biomarkers	0.886	0.895	0.895
	All	0.886	0.882	0.895
	CNN+rad	0.871	0.894	0.888
	Bio+rad	0.896	0.893	0.896
	Bio+cnn	0.874	0.883	0.889

## Appendix B

We have applied CBM to the Lungx Challenge [37] to check whether our approach generalizes to an independent dataset. The Lungx Challenge consists of chest CT scans with annotated nodule centroids and malignancy status. This dataset is substantially different from and more challenging than the LIDC dataset. The training set consists of just 10 patients with 1 nodule each, and the test set consists of 60 patients with 73 nodules in total. Radiomics features cannot be extracted since the Lungx dataset does not come with the required nodule segmentation masks.

The dataset has been preprocessed in the same way as the LIDC dataset (see Section 3). Using the CBM pre-trained on LIDC, we extracted the same biomarkers as before and used them as input features. We found that the Lasso classifier performed the best with an AUC = 0.674, with the regularization parameter being determined via three-fold cross-validation on the whole dataset. Compared to the performance metrics from applying on LIDC, this AUC is low. However, out of 10 groups participating in the Lungx Challenge, the best method (support vector regressor) also achieved a comparable AUC of 0.68. This shows that the features obtained by the CBM generalize to other datasets with competitive performance on the downstream classification task.

## Appendix C

The performance of our model lags behind some of the best black-box models trained on the LIDC-LDRI data, particularly [15] (acc = 0.927) and [17] (acc = 0.953). While the ConRad model focuses on interpretability, we have also explored how to improve its accuracy. The gap in accuracy could be narrowed without sacrificing interpretability by improving the prediction of biomarkers through further refinement of the model architecture, collection of more CT scans, and improvement to the training processes.

As a proxy for improved biomarker prediction, we replaced some of the predicted biomarkers with annotated biomarkers (ground truth). This can increase the overall accuracy up to 0.927 (Figure A1), highlighting the potential for biomarker prediction improvement.
